# “Being Holistic Is a Lot to Ask”: A Qualitative, Cross-National Exploration of Occupational Therapists' Perceptions and Experiences of Holistic Practice

**DOI:** 10.1155/2023/2432879

**Published:** 2023-10-18

**Authors:** Mona Asbjørnslett, Lisebet S. Skarpaas, Linda Stigen

**Affiliations:** ^1^Department of Rehabilitation Science and Health Technology, Faculty of Health Sciences, Oslo Metropolitan University, Norway; ^2^Department of Health Sciences in Gjøvik, Faculty of Medicine and Health Sciences, Norwegian University of Science and Technology, Norway

## Abstract

Being holistic is often used by occupational therapists to describe their practice and philosophy worldwide. This study explores the perspectives of 33 occupational therapists, working in 13 different countries, on their understanding of holistic epistemology and practice and how they seek to incorporate holism in their work. On the basis of a qualitative study design, individual interviews were conducted with the participants by 18 Norwegian undergraduate occupational therapy students, supported by their supervisors. The authors subsequently analyzed the transcribed data, using a thematic analysis approach. Three principal themes emerged: (1) holism as a broad and narrow concept, (2) being holistic spans from treating body parts to teaching marginalized children, and (3) being holistic is a lot to ask. When talking about holism and holistic practice, participants described their holistic practices in various ways, and their accounts reflected different understandings and cultural contexts. Participants characterized a holistic approach as one emphasizing the importance of occupations and activities and helping patients regain independence in their everyday lives. However, they also highlighted the specific challenges they faced, including cultural factors and inadequate resources. Significantly, participants from both Western and non-Western contexts emphasized the importance of holistic practice, suggesting that a dichotomous understanding of Eastern versus Western philosophical approaches does not necessarily make sense in occupational therapy interventions. Therapists' degree of commitment to client-centered practice appears of greater relevance. With its international perspective, our study sheds light on important areas of contemporary occupational therapy practice, including the difficulties occupational therapists face when seeking to cover “everything” in an effort to be more holistic.

## 1. Introduction

Holistic approaches and epistemology are featured in the practice of occupational therapy in different parts of the world. In this study, we define epistemology as a specific way of knowing the world and how knowledge is constructed [1]. As such, clinical practice in occupational therapy is constructed around a core value of being holistic, which is widely present in the occupational therapy literature. For example, in a USA-based national survey, more than 80 percent of occupational therapists using the Model of Human Occupation (MOHO) in their practice reported that this model supported holistic, occupation-focused, client-centered, and evidence-based practice [2]. Research involving Swedish occupational therapists suggests that these practitioners distinguish between a holistic, socially oriented approach on health and a biomedical one [3]. A study from the UK [4] explored how occupational therapists aspired to be holistic and claimed a holistic perspective as a part of their identity and self-definition. However, these therapists often struggled to define the meaning of holism. As they negotiated the tension between theory, beliefs, and practice, they found that holism could at times appear “an illusion” [4].

Historically, holism is an idea that occupational therapists often use to describe and characterize their practice: one where the individual is viewed as an integrated being [5]. In the late 1980s, groundbreaking research by Cheryl Mattingly, an occupational therapist, and Maureen Hayes Fleming, an anthropologist, explored the thought processes of occupational therapists when treating patients and what they thought about their practice, language, and values [6]. As the researchers noted, “occupational therapists have always presented themselves as concerned with the patient's relationship with the disease, as well as with the ‘whole person'” ([6], p.74.). On the basis of our own experience of the relationship between disability and meaningful occupations, we share the view that occupational therapists seek to treat the whole person [6].

For Hasselkus and Dickie, a holistic view of disability differs sharply from a medical understanding. With reference to McColl, she emphasizes that, from a holistic view, disability can be seen as normal experiences of life and with opportunities for personal growth, development, and a unique way of being in the world. From this, it follows that the task becomes one of achieving a balance and living one's life within the constraints of the disability [5, 7].

Holistic ideas and approaches are also evident in recent occupational therapy literature, including that relating to occupation-focused medical rehabilitation [7, 8]. In the book edited by Huri [8], holistic influences are evident in studies that argue for the unique quality of occupational therapy and suggest that a holistic approach enhances patients' life skills to enable participation in everyday life activities [9]. For instance, in her study on psychomotor therapy, Probst employs a holistic view of a unified body and mind, with a focus on movements, in her exploration of the treatment of patients with severe mental health disorders [10].

Akyruek and Bumin [11], whose research focuses on community participation for people with disabilities, refer to Hussey et al. [12] and argue that a holistic approach in occupational therapy involves the following:

*The handling of occupational therapy in all directions of humankind is called holistic approach. Holistic approach emphasizes the organic and functional relationships between whole and parts. This approach assumes the person as a whole as biological, psychological, sociocultural and spiritual*. ([11], p. 83)

Recent literature also emphasizes the central role played by meaningful activity and occupations within the profession. Fisher and Martella argue that a person-, environment-, and activity-centered approach lies at the root of occupational therapy. They stress the importance of understanding occupation as a transactional whole in which occupational and situational elements are always intertwined [13].

The value of holistic approaches has been highlighted by research in other fields, where dichotomous understanding of body and mind is addressed. For example, a study of cancer survivors suggests that mindful engagement in everyday occupations can help such individuals unlock their core self and enjoy enhanced quality of life [14]. It has also been found that to achieve holistic mind-body connection and well-being for women who have suffered childhood abuse, mindful engagement in daily activity can promote a “journey to wholeness” [15].

A persistent claim made in the literature is that there is a gap between Western approaches to healthcare (often characterized as “reductionist”) and Eastern perspectives judged to be more holistic. Tsipris seeks to narrow this perceived gap by advocating the introduction of yoga to an occupational therapy program [16]. Twinley questions the adequacy and holistic depth of the terminology used by therapists, notwithstanding our tendency to view activity and occupations as health-promoting and positive [17].

In general, holistic approaches are sensitive to cultural differences and human diversity; they take into account how people think and feel about health and illness and how they use available health services [18, 19]. Morrison argues for the adoption of a pragmatic epistemology by occupational therapists, one which enables us to see new connections and understand occupations as social phenomena, integral to the way society constructs and reconstructs itself [20].

As a profession characterized by theoretical, ethical, and cultural diversity, occupational therapy can only be enriched by critical perspectives [21]. As Hammell notes, building on our diversity may well enable our work to have a global impact [22]. Despite the presence of holism in occupational therapy literature for several decades, the authors of this study were unable to identify research with an international perspective: that is, research that attempts to explore the ways in which holism is understood and applied by occupational therapists in different parts of the world.

### 1.1. Holistic Philosophy

The premise of our study (project) is that occupational therapy is embedded in an occupation-based and holistic epistemology [23]. For Di Stefano, the holistic philosophy is the basis of complementary and alternative medicine in the West, where being holistic is directed towards understanding a whole system rather than selected events or phenomena. For Di Stefano, being holistic is complementary to biomedical approaches and traditions. Holistic philosophy sees life as influenced by many factors, including those beyond the purview of scientific notions of rationality, materiality, and mechanism [24]. For Di Stefano (citing Jan Christian Smuts), holism has come to signify a philosophical position that acknowledges the central unity of creation. Holistic is a powerful term and carries the synergetic understanding that “wholes are greater than the sum of their parts” ([24], p. XVIII).

With this background, it is evident that the idea of holism has been present in occupational therapy for many years and that it remains a relevant philosophical foundation for our practice. However, we have been unable to locate any previous studies exploring how occupational therapists apply the concept of holism in practice. The research question addressed in the study that forms the basis of this paper is therefore “How do occupational therapists in different practice contexts around the world understand and use the term ‘holistic' in relation to their practice?”

## 2. Design and Methods

The initial stages of this qualitative project were carried out by a team of bachelor students, employing a qualitative descriptive research design [25]. In qualitative descriptive studies, researchers stay close to their data and to the surface of words and events; this is the method of choice when straight descriptions of phenomena are desired [26]. The initial aim of this project was to explore the experiences, reflections, and taken-for-granted ideas of occupational therapists representing different countries and cultures where the meaning of context is particularly relevant [27]. Open, semistructured interviews were conducted with a total of 33 participants, located in a range of different countries.

### 2.1. Participants and Recruitment

The participants comprised 27 female and 6 male occupational therapists, working in Chile, Canada, Australia, Iran, Abu Dhabi, Palestine, Israel, Tanzania, Romania, Germany, the Netherlands, the United Kingdom, and Norway (see Table 1 for participants' demographic characteristics). Three researchers (the authors of this study), together with the undergraduate students involved in this project, used their international networks to recruit participants. The recruitment criteria was that the participants were performing occupational therapy in their present country.

### 2.2. Interviews

Interviews were conducted between 2019 and 2021. Occupational therapy undergraduate students from Norway conducted all 33 interviews as part of their bachelor thesis in occupational therapy. The interviews lasted from 35 to 97 minutes. While some students were able to interview their informants in person, most of them used digital platforms such as WhatsApp, Zoom, and Skype to conduct the interviews. All interviews took the form of life world explorations, described by Brinkmann and Kvale as semistructured interviews. The aim was to illuminate themes of everyday life in an attempt to understand the perspectives of the informants [28]. During the interviews, the students followed an interview guide structured around an open, narrative approach with a focus on events and happenings. They started the interviews with a sequence of open questions, as in the following:

“Can you tell me what happened yesterday at work (a regular weekday)? Let us start with when you came to work. Describe what first happened when you came to work – tell me about your morning routine? Who did you meet? Is that usual?” The students were asked to continue the interviews along these lines, exploring at greater depth activities, situations and/or events, and relationships with other people (positive or negative) that informants saw as central to their occupational therapy practice.

### 2.3. Data Analysis

Initial analysis of the interviews was carried out by a total of 18 undergraduate occupational therapy students for their bachelor degree theses. Seven pairs of students focused on single countries, while a further two pairs analyzed material from two countries. During this primary phase, the focus was on occupational therapists working within specific national contexts.

In order for a subsequent meta-analysis to be carried out, anonymized copies of all transcribed interviews were kept by the principal researchers (the authors of this paper). We then engaged in an inductive, explorative approach to gain an overall view of the topics of concern and taken-for-granted ideas in a thematic analysis, as recommended by Stanley [25].

In the next phase of the analysis, all interviews were uploaded into the NVivo software and coded inductively by adding open codes to participants' words or phrases [25, 27]. During this process, it became clear that holistic ideas and holistic practice were topics which appeared in most of the interviews. Regarding this as a “taken-for-granted idea,” we then returned to the raw material and read it afresh through the lens of holism. During this phase, we also had several discussions on the dimensions of holism and being holistic. On this basis, we highlighted passages which described or offered reflections on this concept.

Text condensation and line-by-line coding were then applied to these passages, so that we could build codes inductively and evaluate in detail participants' statements related to the holistic approach.

In the third phase of the analysis, similar codes were brought together to form categories [25]. The categories were divided into holistic epistemology and holistic practice. We further discussed the coding and categories and agreed on three main themes: (1) being holistic as a broad and narrow concept, (2) being holistic spans from treating body parts to teaching marginalized children, and (3) being holistic is a lot to ask. Through further analyses and abstraction of the results, the authors of this paper were inspired to develop a conceptual model illustrating the idea of holism as employed and described by the participants. The aim of this visualization was to facilitate the conceptualization of how occupational therapists apply holistic ideas in practice.

### 2.4. Trustworthiness and Validation Strategies

Several methodological concerns about trustworthiness and processes of validation can be raised regarding this study. Firstly, all the interviews were conducted by undergraduate students. It is a strength of the research that the data collection is done by different interviewers, thereby potentially enabling a more broad-based exploration of occupational therapy worldwide than one limited to a single researcher's perspectives. Several of the students involved in the project themselves had a foreign background, enriching the multicultural dimension of the research.

However, according to Brinkmann and Kvale, validation is related to craftsmanship [28]. The undergraduate students lacked research experience, which raises questions about the quality and depth of the data gathered. While some students performed their interviews in English, others interviewed in their native language, subsequently translating the transcription into Norwegian or English. This, too, raises questions regarding the degree to which the results represent an accurate interpretation of participants' meanings [29].

We would argue that our study is strengthened by the richness and diversity of the data collected, enabled by our decision to reach out to occupational therapists across the world: professionals with different educational, cultural, and practical backgrounds and experience. On the other hand, the validity of interview data gathered across countries, cultures, and languages may suffer through loss of accuracy and nuance in cultural or linguistic translation. Given that the students handled both the transcription and translation of the interviews they conducted, linguistic inaccuracies and misunderstandings may have crept in.

To strengthen trustworthiness for the student projects, member checking via supervision, methodological decisions, discussions, and presentations to other students was introduced at the start [25]. To minimize the risk of misunderstanding the content of participants' responses, most students worked in pairs, discussing the process with costudents and also their supervisors. Another criterion of validation is authenticity: the degree to which researchers capture the multiple perspectives and values of their participants [29]. Given the international scope of our study, we would argue that this criterion is met to some extent.

Questions about reliability arise from the fact that the authors of this study conducted only the secondary analysis of the data; they did not carry out interviews and transcribe or interpret the primary data themselves [29]. However, all three authors supervised the students during the research and were very familiar with the research process, thereby strengthening their credibility as researchers [28]. The authors also engaged in a long process of reading the material, coding its content, and discussing and interpreting emerging themes, all of which strengthen the validity of the results [30].

### 2.5. Ethics and Ethical Considerations

Approval for collecting and storing the data was granted by the Norwegian Center for Research Data (project number 100341). The participants were informed that participation in the interviews was voluntary, their responses would be treated in confidence, and there would be no negative consequences from participating in the study. Written informed consent was provided from all participants. All methods were carried out in accordance with relevant guidelines and regulations.

## 3. Results

In the presentation of the results, excerpts from interviews are set in cursive, with the nationality of the participant added, in brackets, at the end of the quote. As they explained their own practice of holistic occupational therapy, their accounts reflected different understandings and cultural contexts.

### 3.1. Holism as a Broad and Narrow Concept

Holism was described and explained in a variety of ways, taking both a broad occupational perspective, such as in community practices, and a narrow understanding, such as in individual physical training, but despite their quite different practices and cultural contexts, the concept seemed to be a central part of occupational therapists self-understandings worldwide.

When discussing the concept and use of holistic approaches, participants spoke of how theories within occupational therapy, such as in the Model of Human Occupation, were associated with holistic perspectives. As participants explained, such theoretical perspectives helped them *take into consideration the environment and not only the personal factors* (Palestine), by taking into account factors beyond the purely medical/physical aspects. In this quote, it is evident that considering the environment was always present in a holistic approach.

Even though not all the participants distinguished holism in occupational therapy from forms of medical treatment, other participants sometimes divided between what they saw as holistic and more focused on a specific injury, diagnosis, or impairment. This latter type of focus was seen to represent a pathological and reductionistic view of clients, rather than the broader, activity-based perspective they themselves employed. Turning to one of the broad explanations of being holistic, one of the informants put it like this:

*I would explain this holistic because we see a full view of the person. And we take more of an approach to look at all aspects instead of a lot of other professions, focus more on the injury, the diagnosis or the impairment, whereas we see the individual as a whole, which includes their environment and their activities or occupations and everything else about them.* (Canada)

Holism also seemed to be included when the therapists explained their skills and expertise when helping people with activity problems. One informant also believed that people outside the profession acknowledge how occupational therapists see and focus on the whole person: the participant put it like this:

*And I think we have the lucky position of being able to understand our client holistically and how we can help them be able to do what they want to do and use our expertise and creativity to help enable them to get back to those activities.* (Australia)

Explanations of holism does necessarily inherit what a full view of a person or looking at all aspects means, but when therapists add the environment, doing, and occupation, this makes more meaning to our specific professional viewpoints. Thus, taking an overly broad or holistic view of our profession may make it more difficult for occupational therapists to explain what exactly it is that they do—and may reinforce views at large in society to the effect that occupational therapy is vague and nebulous.

### 3.2. Being Holistic Spans from Treating Body Parts to Teaching Marginalized Children

A holistic approach was generally seen as one stressing the importance of occupations and activities and helping patients regain independence in their everyday lives. At the same time, holistic treatment and therapy was explained in many ways, depending on social and cultural understandings, as well as possibilities within different countries. Since most participants worked in hospitals or other medical facilities (including private clinics), their perspective on holistic practice tended to be influenced by the health systems operating in their particular country. In addition, the context in which rehabilitation treatment unfolded (in many cases clinics and hospitals) had an impact on individual treatment.

Some participants argued that holistic occupational therapy meant treating individual body parts, while others were concerned with the way specific social and cultural understandings of disability and illness influenced their practices. In Norway, as an example, rehabilitation treatment and assistive technology are paid for by the government, where occupation-based intervention towards the goal of enabling clients to live independently in their own homes is an important aim. However, elsewhere in the world, occupational therapy treatment and assistive technology could be less of a priority. In Iran, for instance, rehabilitation treatment did usually not receive government funding, leaving many people unable to afford assistive technology or therapy. With medical doctors presiding over the allocation of occupational therapy, treatment and goals like independent living might be given a lower priority or even described as a waste of resources. One Iranian participant spoke of the emphasis placed on getting patients with cerebral palsy or in stroke rehabilitation to walk again:

*It is expected that the family of a patient who is unable to walk will ask: “when will he start walking?” This is disappointing. In our culture, disabled people are not accepted. A person doing his work while sitting in a wheelchair is not acceptable for the society. This means that the opportunities for disabled people are limited. I always ask clients and their families what they mean by getting well, or healing: what does it mean to them?* (Iran)

In this example, occupation-based values were not always visible in treatment strategies. Some participants problematized, while wishing to be “more holistic,” and felt limited by their specific work context such as the hospital where they were based, or a country representing a marginalized view on disability as treatable or nontreatable.

In Iran, physical training for patients prioritized for occupational therapy was usually associated with intensive individual training to gain independence:

*I always warm up my hands by putting on cream and massaging them. Then we start by moving the joints in all fingers, thumbs included, folding the hand by bending all the fingers. Hand power, such as squeezing a small ball for about five seconds.* (Iran)

Participants working in other parts of the world had different perspectives. Two participants, the first from Tanzania and the second from Germany, saw a holistic approach to rehabilitation as involving adapting therapy to meet the patient's own needs, requests, and understandings of OT:

*What we do is, we make sure that our therapy is going to focus on helping the client's needs. If the client is having a problem with their hand, then we make sure our therapy is based on that.* (Tanzania)

*And if the patient has another problem --he can be here because of his shoulder but has a back pain – I will treat his back, I do not care, it is just like that. Or if he says “my hand hurts today”, I will do something with his hand. If he has a problem with his psyche, I will talk to the patient in a therapy session.* (Germany)

Another participant, such as one who worked in a community health center, and using a family health model, described how she had adopted to a more *holistic practice*. For her, this meant addressing more than the individual person; it involved working with the client's whole family and wider environment.

Other, such as in a case from Romania, occupational therapists followed up on everyday occupational needs for marginalized children during the COVID-19 pandemic lockdown, where occupational therapists together with other professionals arranged for online schooling in order to prevent the children from dropping out of school. In this example, the occupational therapists used their creative skills not simply to meet children's educational needs but also to help children acquire what the therapists called life and recreational skills, such as serving food and learning about hygiene, where helping children live independent every day is visible through addressing occupational problems.

As a last point in this part, it is important to stress that occupational therapists not necessarily find it satisfying to work only in the environment, because their expertise is also identified with individual treatment approaches, which is problematized from Chile, from a therapist working with children with autism and sensory integrative problems:

*Well, another challenge is that it's difficult to withdraw myself from wanting to do clinical treatment. Wanting to take a child out of the classroom and be able to do a session with him, because sometimes I feel that I have the time. But what is required of me is something else. I don't take a child out of his classroom to do an individual sensory integration session with him, even though I would love to have a ball pool or swings to work with. To be frank, I'm not cut out to work in a purely clinical way.* (Chile)

Like in this quote, participants' accounts of holistic practice within occupational therapy included diverse social and cultural understandings, where they also touched on the limitations and possibility characteristic of communities, hospitals, and rehabilitation centers in their abilities to perform holistic occupational therapy, which was explained as both treating the individual person and working in a broader environment.

### 3.3. Being Holistic Is a Lot to Ask

Some of the participants questioned if our profession is holistic or if the idea of being holistic is too much to ask. Several participants did in fact speak of the difficulty of being holistic in their practices. One informant reflected on the complex foundations of occupational therapy, noting that being holistic, covering a lot, becomes difficult, because occupational therapy needs to adapt to a particular context and then narrows what occupational therapy is about:

*We have to include the whole person, as we are used to doing as OTs. But we also have to be good at* not treating *the whole person, as that is the responsibility of all the community services.* (Chile)

For the participants, occupational therapy principally involved thinking and acting from an occupation-based perspective. While being holistic could be “a lot to handle,” informants agreed that ideas about how occupations affect our lives formed the core of occupational therapy.

Being holistic, however, was trying to see what the patient needs and educate the family and the caregiver about the recourses that can help the patient on things and that may make his life better, make his life easier, and improve life, for example. Working with a patient with a spinal cord injury exemplifies this dilemma:

*It's really difficult to explain to them that this is things that happen when you have a kind of spinal cord cut. You will not gain your old skills before, right now our goal that the wheelchair will be a part of your body, you need to understand that. You need to have a good skill and maneuvering or manipulate the wheelchair, you need to strength the upper body, right now your skills is good you can climb use only your hands, you need to go transfer yourself from the wheelchair down or up or from the wheelchair to the bed and from the wheelchair to the car. Your life will be continued and ok, but there is an extra part added to your life called the wheelchair, you need to understand this.* (Abu Dhabi)

Furthermore, the participant elaborated and continued:

*So when I try to explain this to the patient, the whole goal of the family and the patient is shifted from taking medication, trying to find a solution or waiting for a miracle, to another goal, a functional goal that make his life better -- he's moving on, he's trying to think “I will return to work again, I want to continue my life, to have my own home, I will get married soon”. So this is a kind of shifting the goals or trying to broaden the patient's tunnel vision … this kind of feeling I believe in more than other theory.* (Abu Dhabi)

Questioning the wide-ranging expectations, including families and patient surrounding their work as occupational therapists, participants observed that attempting to apply a holistic approach could make life even more difficult for them. They suggested that a holistic approach always needed to be limited to a particular context or situation and that the field in which they worked usually limited their opportunity to treat the whole person.

They also described dealing with multiple challenges on a daily basis, constantly aware of the high expectations surrounding them. While their occupational therapy services often adapted to environmental expectations and community and workplace possibilities, this could create more problems for therapists wishing to be more holistic while at the same time meeting the requirements of real world practice.

As highlighted by a Canadian occupational therapist, *to remain client-centered and know and use those holistic theories and principles of OT, and then balance that with what the hospital is asking you to do in a hospital setting is difficult* (Canada). She further elaborated and problematized being holistic while having to treat patients at a hospital under strict time constraints:

*And I think it can become hard to balance that holistic, client-centered model and then the pressure from the hospital and management and administration to get people out as quickly as possible. And you have very limited time with each person because caseloads are so large it can become overwhelming. So I think it's definitely a balancing act….* (Canada)

As in this case, when the main goals of therapy are being able to go to the bathroom by themselves, get dressed, and make a simple meal, where leisure activities are less important in the hospital setting, being holistic is questioned by the therapist. The participant problematized how hospital settings and a medical context could make it difficult to follow patients' own needs, or being holistic and client-centered. An occupational therapist working in Gaza also gave an example of what happened when a client's individual priorities were overlooked during rehabilitation:

*The client told me:“You did me lot of good and I learned how to eat, how to dress, how to transfer, how to move with a chair, and it is very good. But I don't even know how to do my prayers, how to go to the mosque, you know, for Friday prayers, how to perform this.”* (Palestine).

As suggested above, many participants identified the attitude of being client- or family-centered as part of holistic practice. Being client-centered was seen to involve meeting clients where they were at a particular moment and addressing their priorities, interests, and needs.

As the participants pointed out, individual training may not necessarily conform with holistic practice, particularly where therapists are required to work within many different domains. In such situations, was working holistically too much to ask?

## 4. Discussion

The aim of this study was to explore the perspectives of occupational therapists in different parts of the world on holism and being holistic in their everyday practice. Three main themes emerged from the analysis of interview data: (i) holism as a broad and narrow concept, (ii) being holistic spans from treating body parts to teaching marginalized children, and (iii) being holistic is a lot to ask.

**Figure 1:**
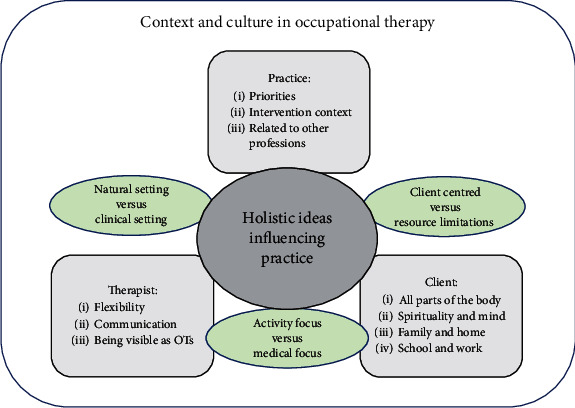
Conceptual model of participants' descriptions of holistic occupational therapy practice.

In Figure 1, we bring together in the form of a conceptual model the various perspectives provided by our participants on what holism is, how it is practiced, what it takes from the therapist, and the challenges and dilemmas related to holistic occupational therapy.

The participants' strong expressions of the perception of holism as a core concept in occupational therapy place holism at the center of our model. Furthermore, the participants' value of holism extended beyond occupational therapy literature and practice to wider society and culture. When considering holism in relation to their clients, the participants offered a variety of perspectives, whether related to body and mind or to the different arenas in which clients lived their everyday lives. Their ideas, summarized in the client square at the bottom right side of the model, are captured in the theme “holism as a broad and narrow concept.”

Participants' reflections on what holism demands of them as therapists, including the skills required of them, are summarized in the therapist square at the bottom left side of our model and are presented in greater detail in the theme “being holistic is a lot to ask.”

Participants also provided a range of perspectives on how holism related to their practice as occupational therapists. The square labelled “practice” at the top of our model presents some broad areas mentioned by participants in relation to their practice, including challenges to holistic work posed by their specific work context and the complexity of applying a holistic approach when working in different settings. This is described in the theme “being holistic spans from treating body parts to teaching marginalized children.”

Separating the three large squares circling our model's core theme are three smaller elliptical bubbles, shaded green. These represent three dilemmas or conflicting viewpoints that the participants seem to juggle in their practice. Firstly, there is the question of whether to practice in a clinical setting or a more “natural” one, such as the client's home. Secondly, therapists may find themselves having to choose between a strictly medical focus and an approach more geared to activity and occupation. Thirdly, therapists are confronted by the gap between their desire to be more client-centered and the limited resources at their disposal. These dilemmas represent some of the tensions experienced by the participants regarding their holistic practices, as captured in the theme “being holistic is a lot to ask.”

### 4.1. Findings in relation to the Existing Literature

Our themes illustrate and shed light on the ways in which occupational therapists in different parts of the world understand the term “holism” and the concept of “being holistic.” In general, their perspectives reflect those found in occupational therapy literature [6–8]. Their descriptions also accord with the findings of studies [3–5, 31] which highlight how holism is frequently used to describe and characterize occupational therapy practice and thereby to inform practice.

When describing what they understand by the meaning of “being holistic,” occupational therapists tend to use broad, even lofty, definitions. They talk about their efforts to be “holistic thinkers” and capable of “seeing and treating the whole person.” This in turn raises questions such as what does it mean to “see the whole person.” One answer suggested by the participants is having the aim of helping people do what they want to do. This is consistent with what is generally understood as client-centered occupational therapy: working closely with clients and having respect for their preferences, values, and choices [13].

Our themes, particularly the second one, illustrate the diversity of settings in which occupational therapists seek to apply holistic approaches. These attempts unfold in family homes and local communities; they can extend to school-based work with marginalized children requiring help with basic life skills. There are similarities here with an occupation-based understanding of working in tandem with clients who are actively engaged in real occupations [13]. Here, cultural variations emerge as significant. While some countries and cultures emphasize the importance of independent living, others may not see this as the main goal of occupational therapy interventions. This does not mean that occupational therapists working in such contexts do not have a holistic approach but rather that they must operate within broader social parameters (for example, in relation to disability and illness). While some societies may view disability as an opportunity for growth and development [7], it can also be understood as a hindrance to independent living and in some cases not worth treating.

The meaning of occupations in everyday life finds emphasis in occupational therapy literature [6–8, 13]. However, less attention has been paid to what practicing occupational therapy might mean in different contexts: in hospitals versus medical clinics, for example, or in different cultural settings. Perhaps as a result of this, occupational therapists explain holistic therapy in a variety of ways; for our participants, massaging a hand, treating a back pain, and asking a patient to squeeze a ball are all examples of holistic practice. To justify that this can be a holistic approach, we can turn to Di Stefano saying that treatment is judged to be holistic as long as it is connected or complementary to a broader unity [24]. In this case, holistic practice can be seen as something that leads to further functional opportunities for the patients.

Significantly, occupational therapists working in both Western and non-Western societies spoke of the link between physical training and holistic practice. This suggests that a dichotomous understanding of Eastern versus Western philosophy and practices does not necessarily make sense in occupational therapy interventions. More relevant is the commitment to practice that is essentially client-centered. As Fisher and Martella [13] note,

*Client-centered practice requires that occupational therapists develop therapeutic rapport and a collaborative relationship with their clients, and then work with their clients in a manner that is respectful of their clients' own perspectives, preferences, values and choices.* ([13], p. 64)

Our final theme captures the challenge implicit in attempting to “see the whole person,” which is indeed a lot to ask. In Finlay's [4] study, some therapists found the challenge so great as to suggest that holism might be an illusory goal [4]. Across the world, resources available for occupational therapy are limited, suggesting that asking practitioners to cover “everything” in their effort to be more holistic may be unrealistic. Perhaps in some cases, our services would benefit from some degree of narrowing, when working with physical impairment and illness. We end this discussion with a definition, drawn from the literature of medical rehabilitation. For Akyurek and Bumin, a holistic approach involves “the handling of occupational therapy in all directions of humankind” ([11], p. 83). This definition is consistent with our analysis, which underlines the multidimensional character of holism and the fact that a holistic approach includes adapting to diverse social understandings of disability and illness. At the same time, this understanding is vague and its meaning is not clear. This raises the following questions: how helpful is our holistic epistemology of “covering everything” to an understanding of what occupational therapy is about? Does it act to support the work of therapists in the field or does it make their work even more complicated?

### 4.2. Conclusion and Looking Ahead to Future Research

In this study, we have investigated the perspectives of occupational therapists located in different parts of the world regarding their understanding of holism as an epistemology and the ways in which they attempt to apply this philosophy in their work. Our analysis leads us to conclude that holism is a many-faceted and broadly understood term which can be interpreted in a variety of ways, according to cultural and social context, political systems, and economic factors such as resource availability. We look forward to further research on the use of theoretical perspectives within our profession, including core ontological and epistemological ideas of what occupational therapy is all about. We recommend that such researches embrace the performance of occupational therapy in different contexts and cultures.

While not covering all cultures and countries, our study indicates that there are significant variations in occupational therapist's understanding and application of holism. We view our research as a contribution to the ongoing debate around the concept of holism and how we, as occupational therapists, might better incorporate holistic approaches in our practice. If we continue to claim that occupational therapy is a holistic profession, we need to clarify our own understanding of holism—and the extent to which it can inform therapeutic practice across a range of different contexts and circumstances.

## Figures and Tables

**Table 1:** Participants' demographic characteristics.

#	Country of practice	Sex	Age group	Current workplace/type of praxis	Work experience
1	Australia	Female	37	Teaching hospital, neurology section	16 years
2	Australia	Female	30-40	Private practice, mixed range of clients	13 years
3	Australia	Female	30-40	Hospital setting, neurology	12 years
4	Canada	Female	20-30	Inpatient geriatric unit	1 year
5	Canada	Female	20-30	Community-based setting	1 year
6	Chile	Female	20-30	Four rural health centers in the municipality	4 years
7	Chile	Female	20-30	Home-based occupational therapy	1.5 years
8	Chile	Female	20-30	School-based and private sessions in homes	1 year
9	United Kingdom	Female	47	Adult social care, council	24 years
10	United Kingdom	Female	43	Adult social care, council	17 years
11	Iran	Female	29	Rehabilitation clinic	4 years
12	Iran	Female	27	Rehabilitation center, private	3 years
13	Iran	Female	30	Private clinic	5 years
14	Netherlands	Male	20-25	Neurological rehabilitation center	1 year
15	Netherlands	Male	20-30	Hospital setting	1 year
16	Norway	Female	50	Neurological rehabilitation in hospital	27 years
17	Norway	Female	38	Rehabilitation in hospital	15 years
18	Norway	Female	35	Municipality health services	9 years
19	Norway	Female	38	Work-related rehabilitation	12 years
20	Romania	Female	30	Pediatric outpatient, hospital	7 years
21	Romania	Female	36	Day center	7 years
22	Tanzania	Female	30-40	Hospital setting and outpatient	7 years
23	Tanzania	Male	40-50	Hospital setting, community health setting and university	15 years
24	Tanzania	Male	30-40	Hospital setting	4 years
25	Palestine	Male	43	University	9 years
26	Palestine	Female	37	OT center, pediatrics	15 years
27	Israel	Female	41	Early development center,	19 years
28	Palestine (Gaza)	Male	54	Rehab hospital	27 years
29	Palestine	Female	39	Physical disability, pediatrics center	17 years
30	Abu Dhabi	Male	38	Outpatient clinic	14 years
31	Germany 1	Female	30-40	Private clinic	15 years
32	Germany 2	Female	30-40	Private clinic	12 years
33	Germany 3	Female	30-40	Polyclinic rehabilitation	2.5 years

## Data Availability

Data is available on reasonable request.

## References

[1] Stanley M., Nayar S., Nayar S., Stanley M. (2015). Deepening understandings. *Qualitative Research Methodologies for Occupational Science and Therapy*.

[2] Lee S. W., Taylor R., Kielhofner G., Fisher G. (2008). Theory use in practice: a national survey of therapists who use the model of human occupation. *The American Journal of Occupational Therapy*.

[3] Björklund A., Svensson T., Read S. (2006). Holistic and biomedical concepts of health: a study of health notions among Swedish occupational therapists and a suggestion for developing an instrument for comparative studies. *Scandinavian Journal of Occupational Therapy*.

[4] Finlay L. (2001). Holism in occupational therapy: elusive fiction and ambivalent struggle. *The American Journal of Occupational Therapy*.

[5] McColl M. A. (1994). Holistic occupational therapy: historical meaning and contemporary implications. *Canadian Journal of Occupational Therapy*.

[6] Mattingly C., Fleming M. H., Mattingly C., Fleming M. H. (1994). Clinical reasoning. *Clinical Reasoning, Frms of Inquiry in a Therapeutic Practice*.

[7] Hasselkus B. R., Dickie V. A. (2021). *The Meaning of Everyday Occupation*.

[8] Huri M. (2017). *Occupational Therapy - Occupation Focused Holistic Practice in Rehabilitation*.

[9] Abaoğlu H., Cesim Ö. B., Kars S., Çelik Z., Huri M. (2017). Life skills in occupational therapy. *Occupational Therapy, Occupation Focused Holistic Practice in Rehabilitation*.

[10] Probst M., Huri M. (2017). Psychomotor therapy for patients with severe mental health disorders. *Occupational Therapy, Occupation Focused Holistic Practice in Rehabilitation*.

[11] Akyurek G., Bumin G., Huri M. (2017). Community participation in people with disabilities. *Occupational Therapy Occupation Focused Holistic Practice in Rehabilitation*.

[12] Hussey S. M., Sabonis-Chafee B. (2008). *Introduction to Occupational Therapy*.

[13] Fisher A. G., Martella A. (2019). *Powerful Practice- A Model for Authentic Occupational Therapy Center for Innovative OT Solutions*.

[14] Sleight A., Clark F. (2015). Unlocking the core self: mindful occupation for cancer survivorship. *Journal of Occupational Science*.

[15] Ratcliff E., Farnworth L., Lentin P. (2002). Journey to wholeness: the experience of engaging in physical occupation for women survivors of childhood abuse. *Journal of Occupational Science*.

[16] Tsipris M. O. (2018). *Incorporating the Ancient Wisdom of Bhrigu Yoga into Occupational Therapy Education: The Global-Holistic Occupational Therapy Course*.

[17] Twinley R. (2013). The dark side of occupation: a concept for consideration. *Australian Occupational Therapy Journal*.

[18] Darawsheh W. B. (2015). Towards culturally competent professional practice: exploring the concepts of independence and interdependence. *Research, Policy and Planning*.

[19] Kinébanian A., Stomph M. (2010). Diversity matters: guiding principles on diversity and culture. *World Federation of Occupational Therapists Bulletin*.

[20] Morrison R. (2016). Pragmatist epistemology and Jane Addams: fundamental concepts for the social paradigm of occupational therapy. *Occupational Therapy International*.

[21] Castro D., Dahlin-Ivanoff S., Mårtensson L. (2014). Occupational therapy and culture: a literature review. *Scandinavian Journal of Occupational Therapy*.

[22] Hammell K. (2019). Building globally relevant occupational therapy from the strength of our diversity. *World Federation of Occupational Therapists Bulletin*.

[23] Cutchin M. P., Dickie V. A., Cutchin M. P. (2013). Transactional perspectives on occupation: an introduction and rationale. *Transactional Perspectives on Occupation*.

[24] Di Stefano V. (2006). *Holism and Complementary Medicine: Origins and Principles*.

[25] Stanley M., Nayar S., Stanley M. (2015). Qualitative descriptive, a very good place to start. *Qualitative Research Methodologies for Occupational Science and Therapy*.

[26] Sandelowski M. (2000). Whatever happened to qualitative description?. *Research in Nursing & Health*.

[27] Tjora A. (2021). *Kvalitative forskningsmetoder*.

[28] Brinkmann S., Kvale S. (2015). *InterViews- Learning the Craft of Qualitative Research Interviewing*.

[29] Creswell J. (2007). *Qualitative Inquiry & Research Design- Choosing among Five Approaches*.

[30] Kvale S., Brinkmann S. (2009). *Interviews- Learning the Craft of Qualitative Research Interviewing*.

[31] Nayar S., Stanley M., Nayar S., Stanley M. (2015). *Qualitative Research Methodologies for Occupational Science and Therapy*.

